# Commentary: Neural Changes Associated with Treatment Outcome in Children with Externalizing Problems

**DOI:** 10.3389/fpsyt.2016.00161

**Published:** 2016-09-26

**Authors:** Timothy R. Rice

**Affiliations:** ^1^Icahn School of Medicine at Mount Sinai, New York, NY, USA

**Keywords:** emotion regulation, defense mechanisms, oppositional defiant disorder, executive function, psychoanalytic therapy

## Introduction

In a 2011 study published in the journal *Biological Psychiatry*, Steven Woltering and colleagues demonstrate that children with externalizing behaviors who respond to cognitive behavioral therapy show electroencephalogram-defined neural markers of improved self-regulation (1). In this commentary, I review this study and the rationale for my proposal that children with externalizing behaviors who respond to psychoanalytic psychotherapy may also show similar neural markers of improved self-regulation. The intent is to help psychoanalytically oriented providers, who may have reservations toward evidence-based medicine and mainstream psychiatric care, to see the value of a brain-based dimensional model of recovery and of hypothesis testing to create an evidence base for practice.

## Definitions and Approaches

From a descriptivist perspective, childhood externalizing behaviors are negative behaviors that are directed toward the *external* environment (2). From the original psychoanalytic perspective that coined the term, these behaviors are the products of children’s tendencies to *externalize* components of developing personality structures onto the external environment (3–5). To a contemporary psychoanalytic perspective, this implicit process serves to reduce internal anxiety and regulate negative affect (6), a component of implicit emotion regulation (7, 8).

While behavioral therapies commonly promote self-regulation through (1) parent management training interventions to reward prosocial behaviors and to extinguish negative behaviors and (2) improving executive functioning through skills training and coaching, psychoanalytic approaches promote emotion regulation through communicating to the child the self-protective meanings of negative behaviors. This intervention alongside developmental help scaffolds the child’s development of alternative emotion regulation capacities (8). As both behavioral therapy and child psychoanalytic psychotherapy target self-regulation through the related constructs of emotion regulation and “hot” executive functions (9), the both modalities’ successful outcomes may show similar underlying neural changes.

## Neural Correlates of Response to Cognitive Behavioral Therapy

Woltering and colleagues’ study provides a model for exploring this hypothesis. This study recruited 140 children aged 8–12 from outpatient treatment agencies with scores on the externalizing scale of the Child Behavior Checklist [CBCL; (10)] within the clinical or borderline-clinical range. These children all received electroencephalography (EEG) while performing a test of self-regulation termed a go/no-go task (11).

All children then engaged in an evidence-based cognitive behavioral therapy program with an integrated parent management training [“Stop Now and Plan”; (12)]. This program entailed 14 weekly 3-h group sessions consisting of parent management training and child interventions. Cognitive restructuring, problem-solving, role-playing, social and token reinforcements, and generalization activities were provided. Groups were led by psychology trainees, social workers, and childcare workers. Supervisors assessed treatment fidelity.

The Child and Adolescent Functional Assessment Scale [CAFAS; (13)] was applied pre- and post-intervention. A pre- and post-intervention decrease of 20 points or more defined a treatment response.

Seventy-one of the original 140 participants completed the treatment with usable data. Fifty-five percent (39 completers) were treatment non-responders, and 45% (32 completers) were responders. Non-responders, responders, and 24 age-matched children recruited from the community then repeated the go/no-go task under EEG.

Treatment responders, but not non-responders, showed reductions in posttreatment relative to pretreatment EEG in needed cortical resources to regulate impulsive behavior during the go/no-go task. Specific findings included, a reduction in the magnitude of the N2 component of the event-related potential (ERP), a defined waveform associated with inhibition of prepotent responses (14, 15). This represents reduced cortical resource requirements for response inhibition, a key executive function. Source space analysis, or analyses to determine the anatomical region from which EEG signals generate (16), pointed to the prefrontal regions associated with self-regulation.

This study suggests that successful as opposed to unsuccessful treatment normalizes aberrant biomarkers of executive functioning in children with externalizing behaviors and are associated with improved executive function performance on a go/no-go task.

## Event-Related Potentials, Executive Functions, and Emotion Regulation

The study of ERPs is an ideal neurobiological modality to bridge the theoretical executive function and emotion regulation literature. Several studies examine ERPs in children with externalizing behaviors as both a neural correlate of emotion regulation (11, 17–19) and of executive functioning (1, 20). This dual conceptualization suggests that psychotherapies that target executive function deficits (1) and emotion regulation deficits (19) operate on the same neural system. This facilitates a harmonization of executive skills coaching and other cognitive behavioral interventions with psychoanalytic psychotherapies in addressing externalizing behaviors: Contemporary psychoanalytic psychotherapy practices are hypothesized to target the implicit emotion regulation system (7).

## Executive Functions, Emotion Regulation, and Psychoanalytic Psychotherapy

Contemporary child psychoanalytic psychotherapy involves the interpretation of children’s defenses against unwelcome affects (6, 21–23). This experience-near technique targets children’s guarding against experiencing painful feelings through automatic recourse to externalizing behaviors. This style is an implicit emotion regulation strategy. Implicit emotion regulation (24, 25) shares neuroanatomical correlates with those of the “hot” executive functions (9). “Hot” denotes automaticity, rapidity, and colored by emotion and include ventral prefrontally mediated automatic and effortless modulation of limbic and visceromotor areas (26). These areas match those in Woltering et al.’s study that were shown to be implicated in responsiveness to behavioral interventions. The sharing of a functional neural system target with behavioral interventions implies what clinicians and theoreticians [e.g., Ref. (27)] have long proposed: all modes of psychotherapy hold much more in common than in difference.

## Exploring This Hypothesis and Conclusion

An 8-week manualized psychoanalytic approach to children aged 6–12 with externalizing behaviors organized around defense analysis (28) is under initial study. This trial may be eligible for evidence testing through neurobiological markers, including ERPs as well as functional magnetic imaging (fMRI). Child psychoanalytic psychotherapy stands to benefit from such study.

## Author Contributions

The author confirms being the sole contributor of this work and approved it for publication.

## Conflict of Interest Statement

The author declares that the research was conducted in the absence of any commercial or financial relationships that could be construed as a potential conflict of interest.
